# Studienstart International of the University of Cologne: The closely supervised semester of study entry for students from third countries using the example of the model degree program for human medicine

**DOI:** 10.3205/zma001206

**Published:** 2018-11-30

**Authors:** Yassin Karay, Katja Restel, Rebecca Marek, Britta Schlüter de Castro

**Affiliations:** 1 University of Cologne, Faculty of Medicine, Dean's Office for Student Affairs, Cologne, Germany; 2 University of Cologne, Department of International Affairs, Division 92 International Students, Cologne, Germany

**Keywords:** study dropout, international students, integration, study entry, student numbers

## Abstract

**Objective: **Due to the increasing degree of internationalization and migration movements, German universities must increasingly pay attention to the cultural diversity of their students. Numerous studies have shown that there is still considerable room for improvement at German universities with regard to the integration of foreign students. Therefore, the University of Cologne aims at improving study conditions for students from third countries (especially non-EU) by introducing a compulsory study entry semester *Studienstart International*. In this study, we ask if effects on the dropout rates of international students before the introduction of *Studienstart International* can be observed after the introduction of the study entry semester. In addition, results of a survey of foreign students about familiarizing themselves with the German university educational system and on social contacts with fellow students are presented.

**Methods: **After a one-year voluntary pilot phase in summer semester 2009 and winter semester 2009/10,* Studienstart International* was made compulsory for Non-EU students in preparation for the study of human medicine. 113 students of the human medicine degree program have taken part in this program since the beginning of winter semester 2016/17. The comparison of dropout rates before and after the introduction of *Studienstart International* as well as the results from a survey of foreign students are presented descriptively. Statistical significance of the difference in dropout rates between the two groups of students is tested using a Chi^2^-test.

**Results: **The dropout rates of foreign students fell slightly from 19% to 14% following the introduction of the mandatory study entry semester, but this difference is not statistically significant (Chi^2^=0.785, df=1, p=0.376). According to the survey, almost three-quarters of foreign students have contact with German fellow students at the university, 55% of whom report daily contact. 68% of respondents have contact with German fellow students outside the university. 97% feel they are well prepared for the degree course and 91% say they know where to go with questions and problems during their studies.

**Conclusion: **With the introduction of the compulsory study entry semester *Studienstart International*, the University of Cologne aims at improving integration of foreign students. Although dropout rates could not be significantly reduced, survey results suggest that *Studienstart International* can certainly support international students. Reliable statements on the success of the study cannot yet be made due to the small number of cases. A follow-up study will deal with this question.

## Zusammenfassung

**Zielsetzung: **Auf Grund des steigenden Internationalisierungsgrads und der gesellschaftlichen Entwicklungen im Hinblick auf Migrationsbewegungen müssen deutsche Hochschulen verstärkt die kulturelle Vielfalt ihrer Studierenden im Blick behalten. Zahlreiche Studien belegen, dass an deutschen Hochschulen noch erheblicher Verbesserungsbedarf hinsichtlich der Integration von ausländischen Studierenden besteht. Die Universität zu Köln hat sich deshalb zum Ziel gesetzt, die Studienbedingungen für Studierende aus Drittländern (speziell Nicht-EU) durch die Einführung eines obligatorischen Studieneinstiegssemesters *Studienstart International* zu verbessern. Im Rahmen dieser Untersuchung wird der Frage nachgegangen, ob sich vor der Einführung von *Studienstart International* beziehungsweise nach der Einführung des Studieneinstiegssemesters Effekte auf die Abbruchquoten von internationalen Studierenden nachweisen lassen. Zudem werden die Befragungsergebnisse der ausländischen Studierenden zur Orientierung im Studiensystem und zum sozialen Kontakt zu Mitstudierenden vorgestellt.

**Methodik:** Nach einer einjährigen und freiwilligen Pilotphase im Sommersemester 2009 und Wintersemester 2009/10 wurde *Studienstart International* zur Vorbereitung auf das Studium der Humanmedizin verpflichtend eingeführt. Seit der Einführung haben bis zum Wintersemester 2016/17 113 Studierende des Studiengangs Humanmedizin am Programm teilgenommen. Der Vergleich der Abbruchquoten vor und nach der Einführung von *Studienstart International* sowie die Befragungsergebnisse der Studierenden werden deskriptiv dargestellt. Der Unterschied der Abbruchquoten zwischen den beiden Studierendengruppen wird mit Hilfe des Chi^2^-Tests gemessen.

**Ergebnisse: **Die Abbruchquoten der ausländischen Studierenden sind mit Einführung des obligatorischen Studieneinstiegssemesters von 19% auf 14% leicht gesunken, jedoch ist diese Differenz nicht statistisch signifikant (Chi^2^=0,785; df=1; p=0,376). Laut Angaben in der Befragung haben fast drei Viertel der ausländischen Studierenden Kontakt zu deutschen Mitstudierenden an der Universität, davon geben 55% an, täglich Kontakt zu haben. Außerhalb der Universität haben 68% der Befragten Kontakt zu deutschen Mitstudierenden. 97% fühlen sich durch das Programm gut auf das Fachstudium vorbereitet und 91% geben an zu wissen, an wen sie sich bei Fragen und Problemen während des Studiums wenden müssen.

**Schlussfolgerung: **Mit Einführung des obligatorischen Studieneinstiegssemesters *Studienstart International* verspricht sich die Universität zu Köln eine Verbesserung der Integration von ausländischen Studierenden. Obwohl die Abbruchquoten nicht signifikant gesenkt werden konnten, lassen die positiven Befragungsergebnisse vermuten, dass der *Studienstart International* die internationalen Studierenden durchaus unterstützen kann. Verlässliche Aussagen zum Studienerfolg können aufgrund der geringen Fallzahlen noch nicht getroffen werden. Eine Folgestudie wird sich mit dieser Frage beschäftigen.

## 1. Introduction

International students show increased interest in studying in Germany. As a result of the Bologna Process and the associated joint internationalization strategy of the Federal and State Governments in 2013, the number of foreign students at German universities has risen steadily in recent years [1]. According to the Federal Statistical Office, in 2014 for the first time more than 300,000 foreign students studied at German universities. The declared goal of the Federal and State Governments to enable at least 350,000 foreign students to study at German universities by 2020 was achieved early in the winter semester 2016/17 [2]. Germany is now one of the most popular university locations for foreign students after the USA and Great Britain [1].

Despite these encouraging developments, for example, the survey results of the Deutsches Studentenwerk (Organization providing social, financial and cultural support services to students) from 2012 on foreign students in Germany show that there is still room for improvement in the integration of international students [3]. Especially the search for accommodation, contact with German students and the wider population as well as finding their way around the German university education system still cause difficulties for many international students. Many of the respondents also mentioned struggling with communicating in German. Further studies show that in addition to study-specific factors, the social integration of international students is an important factor in academic success [4]. For example, international students are less likely to consider dropping out of university if they find an inclusive environment at university, and are better able to cope with the study requirements [5]. A study by the German Center for Higher Education and Science Research (DZHW) shows that the dropout rate for foreign students attending a bachelor’s program is 41%, compared to 28% for those with a German school leaving certificate [6]. Regarding medical degree programs, international studies also show higher dropout rates among international students [7], [8]. Furthermore, they show that foreign students spend significantly more time at university before graduating successfully [9]. A study from German-speaking countries shows a significantly longer duration of study until the final exam, especially for non-European medical students [10]. In a 2013 survey of the medical studies deaneries in Germany, 83% of the interviewed representatives rated the German language as the biggest challenge for international students, followed by social integration problems (36%) and intercultural differences (28%) [11].

German education policy is also of the opinion that “teaching a knowledge of German plays a crucial role in the integration and more attention must be paid to language courses offered at universities [1].” According to these statements, universities are called upon to develop approaches, which support the integration of international students into everyday university life. As early as 2009, the University of Cologne responded to the rising numbers of international students and their high dropout rates. It is the first German university, which has started to set up a compulsory study entry semester for third-country students (*Studienstart International*) who wish to complete their entire undergraduate studies at the University of Cologne. Various studies have already confirmed that especially students from Eastern Europe, Asia and Africa have difficulties with the expected behavior and forms of learning at German universities [12]. The Medical Faculty of the University of Cologne also saw the greatest need amongst these student groups.

This article examines the question of whether effects on the dropout rates of international students can be seen before the introduction of *Studienstart International* or after the introduction of the study entry semester. In addition, international students who have passed through *Studienstart International* were asked about their social contact with German and international students as well as about finding their way around the university education system, as these issues are considered the biggest problems in integrating students into their study environment.

## 2. Method

### 2.1. Conditions

The University of Cologne is currently one of the largest German universities with more than 50,000 students. Due to its geographical location and the wide range of fields of study offered, the university is particularly attractive to international students. During the winter semester 2016/17, more than 5,000 foreign students studied in Cologne, and made up approximately 10% of the total student body. The University of Cologne wanted to be the first university in Germany to improve the study conditions for foreign students from third countries by introducing the compulsory study entry semester *Studienstart International*. A primary objective of the study entry semester is not only to guide international students but also to offer them orientation aids and opportunities for participation and integration into their study environment right at the commencement of their studies. This initiative, initially compulsory for medical students, was funded by the DAAD as a PROFIN model project from 2010 to 2013 and since then has been gradually extended to the university’s other faculties under the management of Division 92 - International Students at the University of Cologne. 

#### 2.2. Studienstart International

##### 2.2.1. Legal basis

The regulations regarding proof of ability to study for foreign applicants to the University of Cologne “govern the conduct of the study ability test for foreign students from countries whose university entry qualifications are not considered equal to German qualifications based on international treaties.” Foreign applicants must demonstrate their ability to study at the University of Cologne by taking a study ability test. “In order to start their course of studies at the University of Cologne, the following is required of students:

1. successful participation in the Test for Foreign Students (TestAS) offered by the Society for Academic Study Preparation and Test Development e.V., and
2. successful participation in the practical study examination sections as part of the one-semester program *Studienstart International* at the University of Cologne.”

##### 2.2.2. Curriculum and content

*Studienstart International* lasts at least one semester and directly precedes the degree course. The contents range from various specialist or foundation events in the field of study to subject-specific German courses through to seminars on inter-cultural awareness and study competencies (see Table 1 (Tab. 1)).

At the events for **improving German language skills**, special attention is given to the teaching of German as a scientific language. Students take a grading test so they can receive support according to their existing language skills. It has been shown that the existing knowledge levels of German of students varies widely and that students often have not achieved the level of language competence required for studying at university – despite having passed the DSH (German Language Test for University Entrance). The competence-based German course only has to be completed as part of the program if a student achieved Level 4 or lower in the German grading test. If students do not reach Level 4 during the course of the semester, another pre-semester course in German as a Foreign Language (5-6 weeks) must be completed during the semester break after *Studienstart International*. 

The **Seminar Interkulturelle Sensibilisierung** (Inter-cultural Awareness Seminar) was conceived in order to facilitate the potential of cultural diversity, the students bring with them, to achieve effective and successful cooperation (see also [13], [14]). The seminar is open to students from all faculties and enables international and local participants to meet in a protected space. Exchanges and reflection on values, culture and communication take place under professional guidance. The seminar is interactive and includes topics such as clarification of the concept of culture, cultural models and inter-cultural communication. The connection to relevant inter-cultural encounters (dialog with international patients and colleagues) is emphasized on, and presented in detail through Critical Incidents. In addition to regular and active participation, an essay must be written to fulfill the course requirements.

The **study competence course** provides students with an overview of the relevant forms of communication at university and introduces them to the usual tasks of a student. Getting to know things such as learning strategies and time management strategies enables students to reflect on their behavior in terms of learning and studying, thus develop helpful methods for successful studies. The course, amongst other things, covers topics such as time and self-management at university, exam formats (with particular reference to the multiple-choice exams at the medical faculty) and communication at the University of Cologne (email correspondence with lecturers, etc.). In addition to regular and active participation, a presentation must be held to fulfill the course requirements. 

**Orientation events** and intensive **support** complement *Studienstart International*. This means that students also receive, for instance, assistance in finding accommodation and/or in administrative matters. **Mentoring programs and tutorials**, which delivered by student assistants, flesh out the offers of *Studienstart International*, always with the aim of integrating international undergraduates as early as possible into the overall student body. These include, amongst others, a library tour, study and learning advice, information on scholarship programs through the Central Student Advisory Service, getting to know the AStA, getting to know the advice and social offers of the Cologne Student Union, getting to know the university groups/communities and lectures on the history of the city and the university. Contents specific for the faculty of medicine are also part of the orientation series, such as a visit to the transfusion and anatomy sections, a lecture on the German health system and an introduction to the contents of the pre-clinical part of studies.

The **Tutorial on German terminology** intends to prepare students for the medical terminology practical, which takes place during the first semester in Cologne. While students must pass the subject-specific **biology practical**, the **chemistry practical** is optional. For the biology practical, international students are prepared for this exam in a tutorial and have three attempts to pass the exam as part of *Studienstart International*. When enrolling in the first semester, the subject-specific biology practical and the chemistry practical will only be credited if the students have passed the respective exams. 

International students receive a program of events tailored to their needs and knowledge during *Studienstart International*. They are closely supervised and looked after in *Studienstart International* by staff members of the Department of International Affairs and the faculties, so that in the event of potential problems an early intervention can be made. After successfully completing* Studienstart International*, students will be enrolled directly in the first semester. The prerequisites for admission to the degree program are passing the above-mentioned exams and regular participation in the above-mentioned courses (see Table 1 (Tab. 1), Nos. 1 to 7). Passing the chemistry practical is not a requirement for passing *Studienstart International*. If the students do not pass the study entry semester *Studienstart International* they can once re-take the semester. 

#### 2.3. Sample

After a one-year (optional) pilot phase in the summer semester 2009 and winter semester 2009/10, *Studienstart International* program was introduced as obligatory in the summer semester 2010. Every winter and summer semester approximately nine students are enrolled in the *Studienstart International* program for the human medicine degree course. Overall, since the introduction in the summer semester 2009 until the winter semester 2016/17, N=113 students of human medicine had completed the entry semester. The international students (non-EU countries) of the summer semesters 2005 to the winter semester 2009/10 and who did not complete *Studienstart International*, were used as a comparison group (N=102). The following Table 2 (Tab. 2) summarizes and compares the socio-demographic characteristics of the two groups.

#### 2.4. Procedure

In the course of the investigation, the study progression of 215 foreign students were examined with regard to study dropout (the termination of studies without a degree). Students who have changed university are not included in the statistics, since successful graduation at another university is a possibility. The dropout rates listed in this study are not directly related to interview data to identify reasons for dropping out.

The *Studienstart International* team at the University of Cologne developed the questions asked in the final survey (see Attachment 1). The written survey is voluntary and anonymous and conducted in the last week of the program. Since not all of the questions asked in the survey are useful for the research question of this specific article, only the questions relevant to the study are analyzed. The questions relate to social contacts with fellow students as well as getting their bearings in the university education system. The questions have to be answered with yes or no or on a three-point scale. 

The Chi^2^ test is used to measure the difference between the dropout rates between the students who took* Studienstart* and those who did not, and descriptive statistics are used to evaluate the survey data.

## 3. Result

### 3.1. Dropout Rates

Of the 102 international students who did not go through *Studienstart International*, 19 students (19%) dropped out of medical studies prematurely. While of the 113 international students who graduated from *Studienstart International*, only 16 students (14%) ended their studies prematurely. Although there is a slightly positive trend in terms of dropout rates, this difference is not statistically significant (Chi^2^=0.785, df=1, p=0.376) (see Table 3 (Tab. 3)).

#### 3.2. Final survey after the Studienstart International semester

The paper-based final survey, which is carried out after the *Studienstart International* semester has been completed, by a total of 102 students. Based on the number of students enrolled in the study period (N = 113), this corresponds to a response rate of 91%. A comparison of survey results between international students who have and those who have not completed the entry-level semester cannot be carried out, because the survey was only conducted with the introduction of *Studienstart International*. 

The final survey shows that 74% of these students have contact with German students at university. Of these students, 55% had contact with German students at the university daily or several times a week, 24% once a week and 21% less frequently.

On the question of whether there is contact with German students outside of university life, 68% of the students answered with “yes”. 48% of international students had daily contact. 27% had contact only once a week and 25% even less frequently.

However, contact with other international students was more pronounced. 100% of respondents said they had contact with international students at the university, 88% of whom had regular contact. 10% had contact only once a week and only 2% less frequently.

On the question of whether the students feel well prepared for the degree course, 97% answered with “yes”. Having completed the program, 91% of respondents know to whom they can turn to with questions and problems during their studies. However, the two questions were only answered by a little more than 60 students, since these two questions were only included in the survey from the winter semester 2012/13.

## 4. Discussion

The University of Cologne has introduced a mandatory study entry semester for foreign medical students (*Studienstart International*), with the aim of improving the study conditions through intensive support. The expectation of contributing to a reduction of the dropout rate through the introductory semester, while there appears to be a tendency towards reduction, there is not yet statistical proof, even though well-known issues such as lack of orientation in the university education system, social contact with German students or deficits in German language skills are addressed in the program. The extent to which *Studienstart International*, for example, leads to shorter study duration or an improvement in the exam results of international students could not be analyzed in the context of this study, because the results are not significant due to the small number of cases. However, the positive survey results from international students suggest that *Studienstart International* can certainly support in the problem areas mentioned above.

A lack of orientation in the German education system is often seen as a negative influencing factor on study progress and is still mentioned as one of the main difficulties of foreign students [3], [15]. According to Rech, disorientation in the German education system can be remedied by effective support [5]. As part of the final survey conducted for this study, more than 90% of international students feel well prepared for the program and know whom to approach with questions and problems during their studies, which is considered a success of the program.

Not only the lack of orientation causes many foreign students problems, but many also feel that contact with German students is difficult [3]. In this survey, the majority of international students (74%) state that they have social contact with German students at the university and (68%) outside the university. Overalls, the results should be seen as positive, but they also reveal that there is yet more room for improvement in terms of social contact with German students, because only just under half have contact daily or several times a week. By contrast, daily contact with international students is significantly higher at almost 90% and confirms the observations made by the representatives of the medical study deaneries that international students join together to form permanent groups and circles of friends from similar cultures [11]. This may result in contact with students with a German educational background becoming more difficult. Against this background, supplementing *Studienstart International* with special tandem or mentoring programs between foreign and German medical students should be considered. Mentoring, for example, makes it possible to bring international and German students together, so that a subject-related and inter-cultural exchange can take place on both sides [16].

In order to be able to establish contact with German students in particular, good to very good German skills are essential. In the context of the program, a focus is therefore placed on teaching the German language, because in addition to vague ideas concerning the degree course, a lack of knowledge of the German language can often be a reason that leads to dropout by foreign students [17]. In a survey of the medical study deaneries in Germany from 2013, the majority of the interviewed representatives saw the German language as the biggest challenge for international students [11]. However, the focus must not only be on the linguistic and scientific integration of foreign students into the existing university education system, but also on inter-cultural differences such as different educational experiences, other learning styles but also other forms of communication and contact should be taken into account [18].

Although the results presented in this study show that the introduction of *Studienstart International* did not significantly reduce dropout rates, numerous studies confirm that successful commencement of studies generally contributes significantly to study success [4], [19], [20]. A lack of social integration and identification with the university as well as a deficit of key interdisciplinary qualifications make it particularly difficult for international students to successfully commence their studies [4]. Against this backdrop, the study entry semester, i.e. the period prior to the start of the actual degree course, is of particular importance for foreign students. For this reason, closely supervised study entry should ideally be followed by closely supervised degree studies [21]. After completing *Studienstart International*, this task is taken up by the study advisors of the medical faculty of the University of Cologne. Study progress analyses are carried out regularly after the first semester in order to identify students with low success rates (regardless of nationality). Follow-on and diversity-oriented study guidance aims to determine the reasons for poor study commencement [22]. In dialog with the student, help offers, which relate to the cause of the problems are suggested so that students can complete their studies with the least possible delays. Apart from the mentioned factors, there are of course other factors that the university cannot influence or only to a limited degree. These include, for example, the motivation for studying in Germany, the actual ability to study, where there often is “a large discrepancy between the qualifications acquired abroad and the qualifications required at German universities” [23], study funding or even family issues [5]. 

A limitation of the present study is that the survey of foreign students was not carried out before the introduction of *Studienstart International*. Thus, a comparison of survey results was not possible, which could have provided information about improvement in the study situation of foreign medical students in Cologne before and after the introduction. The questions asked in the final survey were also not validated and have been modified several times since the launch of the program. For this reason, the present study is to be taken at face-value. Also, the concrete reasons for dropping out of the degree course could not be analyzed due to a lack of data. Furthermore, due to the small sample size so far, it was not possible to compare the graduation rates and exam results of foreign students before and after the introduction of the study entry semester. Furthermore, it should be noted that the study is limited to Cologne, although *Studienstart International* is transferable to other faculties and has already been implemented at all faculties at the University of Cologne with the exception of the faculty of law.

## Conclusion

Although the results of this study do not show any significant change in the dropout rates of foreign students, the results presented show a tendency towards improvement and to date align with the desired changes that led to the introduction of the program. Now, it can still be assumed that the concept behind the *Studienstart International* program can assist foreign students to integrate into the German university education system and be transferred to other educational institutions. Due to the small number of cases, the actual effectiveness of the introductory semester on the study success of students could not yet be adequately evaluated and remains a desirable future project. A follow-up study will investigate this question.

## Competing interests

The authors declare that they have no competing interests. 

## Supplementary Material

Muster End of the Semester Evaluation

## Figures and Tables

**Table 1:**
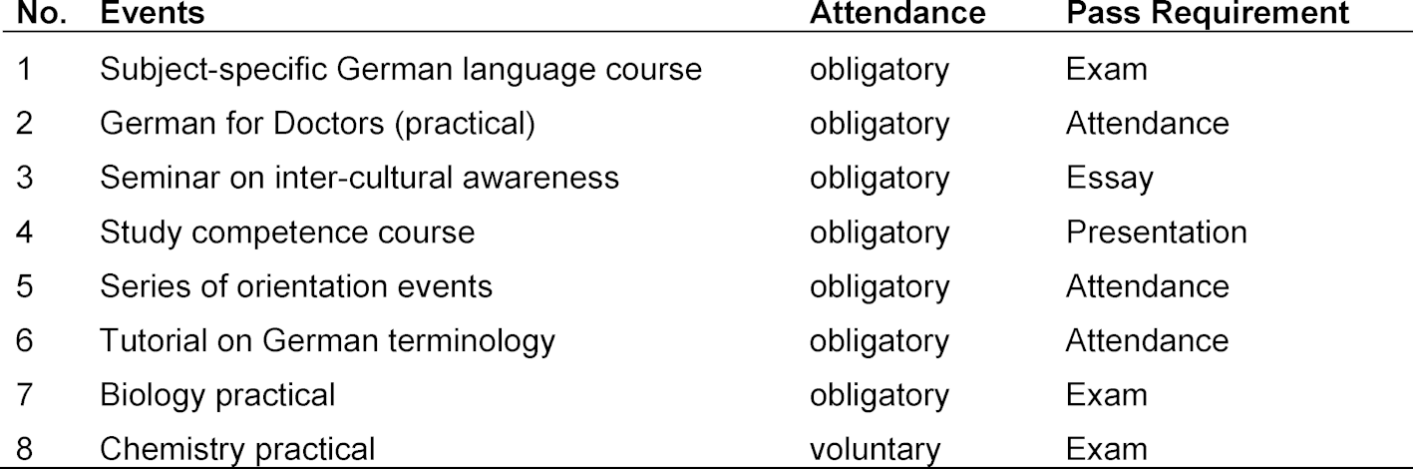
Curriculum for *Studienstart International* for the degree course in human medicine

**Table 2:**
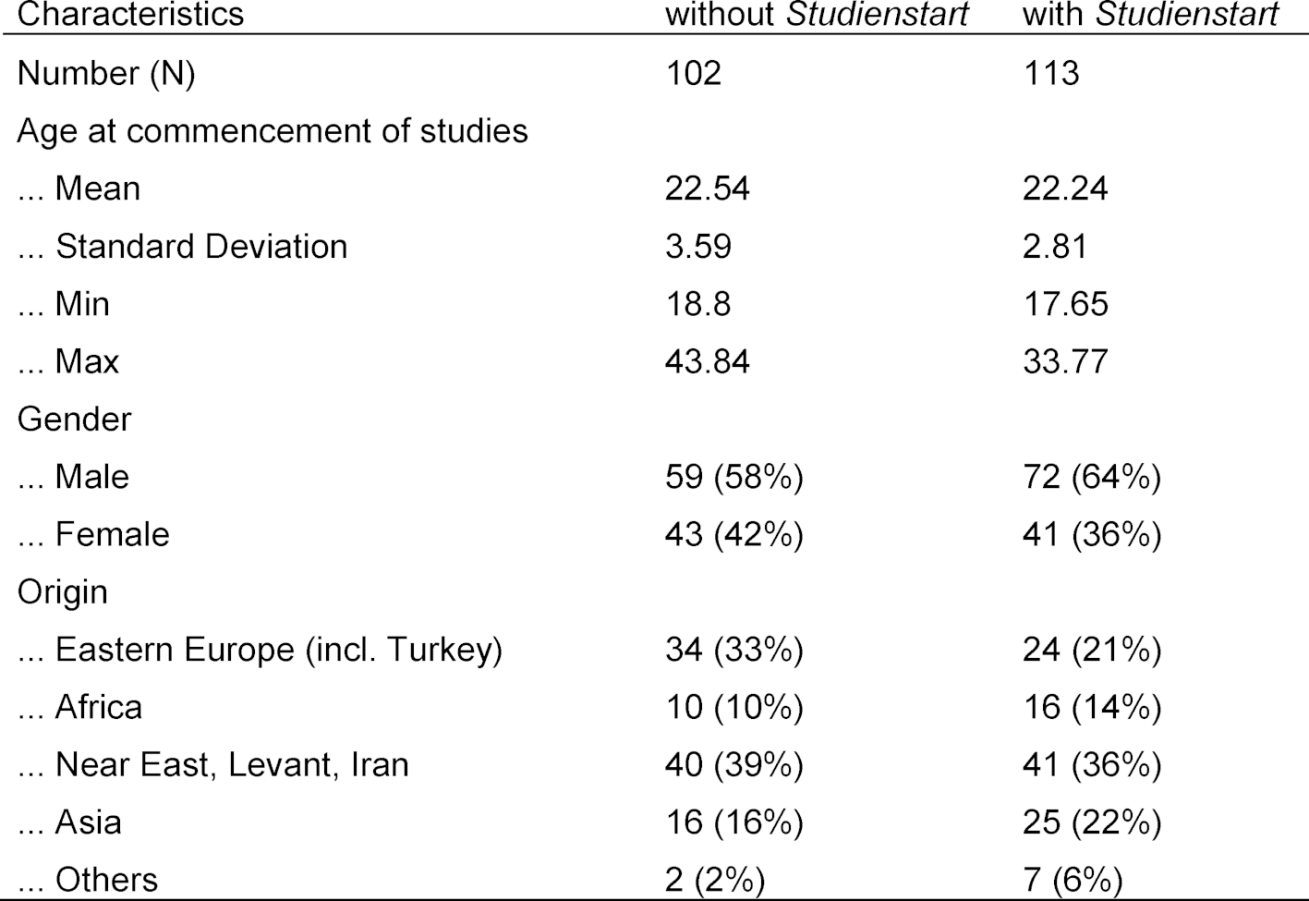
Sociodemographic characteristics of the two groups

**Table 3:**
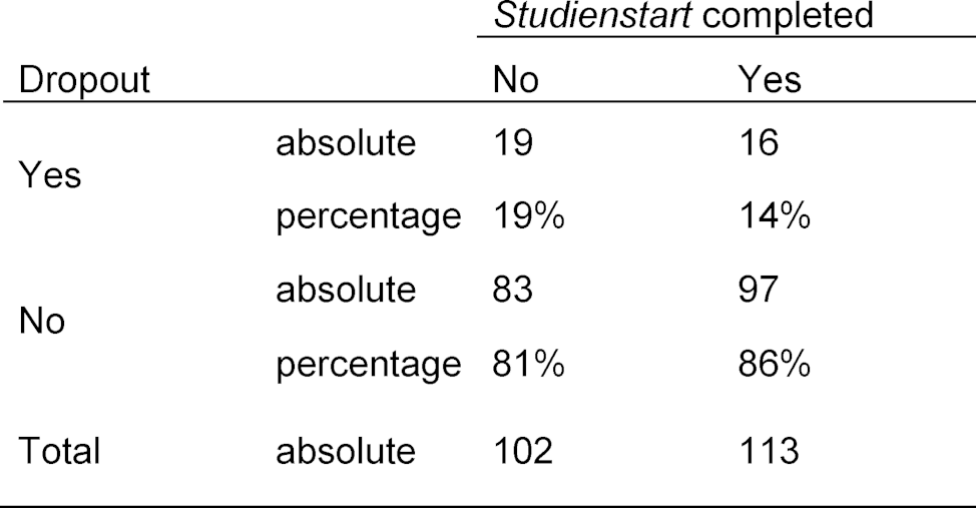
Comparison of the dropout rates of international students who have or have not completed the entry-level semester *Studienstart International*

## References

[1] Bundesministerium für Bildung und Forschung (2015). Pressemitteilung: 098/2015: Erstmals über 300.000 ausländische Studierende in Deutschland.

[2] Statistisches Bundesamt (2017). Anzahl der ausländischen Studierenden an Hochschulen in Deutschland in den Wintersemestern von 2014/2015 bis 2016/2017 nach Herkunftsländern.

[3] Apolinarski B, Poskowsky J (2012). Ausländische Studierende in Deutschland 2012. BAMF. Ergebnisse der 20. Sozialerhebung des Deutschen Studentenwerks durchgeführt vom Deutschen Zentrum für Hochschul- und Wissenschaftsforschung (DZHW).

[4] Heublein, U (2015). Von den Schwierigkeiten des Ankommens. Überlegungen zur Studiensituation internationaler Studierender an deutschen Hochschulen. Neue Hochschule.

[5] Rech J (2012). Studienerfolg ausländischer Studierender: eine empirische Analyse im Kontext der Internationalisierung der deutschen Hochschulen.

[6] Heublein U, Richter J, Schmelzer R, Sommer D (2014). Die Entwicklung der Studienabbruchquoten an den deutschen Hochschulen. Statistische Berechnungen auf der Basis des Absolventenjahrgangs 2012.

[7] Shields PH (1994). A survey and analysis of student academic support programs in medical schools focus: underrepresented minority students. J Natl Med Assoc.

[8] Ferguson E, James D, Madeley L (2002). Learning in practice. Factors associated with success in medical school: systematic review of the literature. BMJ.

[9] Castillo-Page L, Zhang K, Steinecke A, Beaudreau J, Moses A, Terrell C (2005). Minorities in Medical Education.

[10] Huhn D, Resch F, Duelli R, Möltner A, Jazi KK, Amr A, Eckart W, Herzog W, Nikendei C (2014). Prüfungsleistung deutscher und internationaler Medizinstudierender im vorklinischen Studienabschnitt – eine Bestandsaufnahme. GMS Z Med Ausbild.

[11] Huhn D, Junne F, Zipfel S, Duelli R, Resch F, Herzog W, Nikendei C (2015). International medical students – a survey of perceived challenges and established support services at medical faculties. GMS Z Med Ausbild.

[12] Schumann A, Knapp A, Schumann A (2008). Interkulturelle Fremdheitserfahrungen ausländischer Studierender an einer deutschen Universität. Mehrsprachigkeit und Multikulturalität im Studium.

[13] Von Queis, D (2009). Interkulturelle Kompetenz. Praxis-Ratgeber zum Umgang mit internationalen Studierenden.

[14] Gwenn-Hiller G, Vogler-Lipp S (2010). Schlüsselqualifikation Interkulturelle Kompetenz an Hochschulen. Grundlagen, Konzepte, Methoden.

[15] Isserstedt W, Schnitzer K (2010). Internationalisierung des Studiums - ausländische Studierende in Deutschland - Deutsche Studierende im Ausland. Ergebnisse der 19. Sozialerhebung des Deutschen Studentenwerks durchgeführt durch HIS Hochschul-Informations-System.

[16] Heidenreich M, Petersen R, Budde M, Brocke P, Doebert G, Rudack H, Wolf H (2017). Von Fremden zu Vertrauten. Praxishandbuch Mentoring in der Wissenschaft.

[17] Ylönen S (2015). Studienbegleitender und studienvorbereitender Deutschunterricht international: Einführung. Z Interkult Fremdsprach.

[18] Schumann A, Kameyama S, Meyer B (2007). Interkulturelle Fremdheitserfahrungen ausländischer Studierende an einer deutschen Universität. Mehrsprachigkeit am Arbeitsplatz.

[19] Yorke M, Longden B (2008). The first-year experience of higher education in the UK.

[20] Berthold C, Jorzik B, Meyer-Guckel, V Handbuch Studienerfolg.

[21] In der Smitten S, Heublein U (2013). Qualitätsmanagement zur Vorbeugung von Studienabbrüchen. ZFHE.

[22] Karay Y, Hallal H, Stosch C (2018). Untersuchung zur Ermittlung eines stabilen Prognoseparameters zur Detektion von beratungsbedürftigen Studierenden – Realisierung von Chancengleichheit durch eine diversitätsorientierte Studienberatung. GMS J Med Educ.

[23] Heublein U, Schmelzer R, Sommer D (2004). Studienverlauf im Ausländerstudium. Eine Untersuchung an vier ausgewählten Hochschulen.

