# Supplementary material for: Studienstart International of the University of Cologne: The closely supervised semester of study entry for students from third countries using the example of the model degree program for human medicine
Source: GMS J Med Educ. 2018 Nov 30;35(5):Doc60. doi: 10.3205/zma001206 (PMC6326400; doi:10.3205/zma001206)
Supplement: Muster End of the Semester Evaluation [file JME-35-60-s-001.pdf]

MUSTER

EvaSys	SI End of the Semester Evaluation	Electric Paper
International Office		
Studienstart International	End of the Semester Evaluation	

Please mark like this ☐ ☒ ☐ ☐ ☐ Please use a ballpoint pen or a felt pen that is not too strong. This questionnaire is entered automatically.

Correction ☐ ■ ☐ ☒ ☐ In the interest of optimal data collection, please note the information given on the left when filling out.

1. Biographical data

1.1 Which subject do you study?

1.2 How old (in years) are you?

1.3 You are... ☐ female ☐ male

1.4 What is/are your native language/s?

1.5 What is your nationality?

2. Language

On the scale of the European Refrence Frame (A1-C2), how do you estimate your German language skills in

2.1	Hearing	A1	☐	☐	☐	☐	☐	☐		C2
2.2	Reading	A1	☐	☐	☐	☐	☐	☐		C2
2.3	Speaking	A1	☐	☐	☐	☐	☐	☐		C2
2.4	Writing	A1	☐	☐	☐	☐	☐	☐		C2
2.5	Conversations	A1	☐	☐	☐	☐	☐	☐		C2
2.6	How have you been placed at the placement Test from the department DaF in the beginning of Studienstart International?	A1	☐	☐	☐	☐	☐	☐	☐	C2

3. Organizational Commitment to University of Cologne (since WS16/17)

Please rate the statements on your degree of agreement on a scale from 1-5 (1 = Don’t agree at all; 5 = Completely agree)

3.1	I am very happy to spend the entire course of my studies at this university.	Don’t agree at all	☐	☐	☐	☐	☐	Completely agree
3.2	I like to talk about my university with people that don’t study here.	Don’t agree at all	☐	☐	☐	☐	☐	Completely agree
3.3	I could easily feel associated with another university to an equal degree.	Don’t agree at all	☐	☐	☐	☐	☐	Completely agree
3.4	I don’t feel particularly emotionally attached to the university	Don’t agree at all	☐	☐	☐	☐	☐	Completely agree
3.5	This university has a big personal significance for me	Don’t agree at all	☐	☐	☐	☐	☐	Completely agree

MUSTER

3. Organizational Commitment to University of Cologne (since WS16/17)

3.6	Even if I wanted to, it would be very difficult for me to leave this university at this point.	Don't agree at all	☐	☐	☐	☐	☐	Completely agree
3.7	Staying at this university is currently not only a necessity but also according to my wishes.	Don't agree at all	☐	☐	☐	☐	☐	Completely agree
3.8	I think, there is currently no real option for me to seriously consider a change of university.	Don't agree at all	☐	☐	☐	☐	☐	Completely agree
3.9	You don't have to be loyal to your university.	Don't agree at all	☐	☐	☐	☐	☐	Completely agree
3.10	It is ok to change from university to university.	Don't agree at all	☐	☐	☐	☐	☐	Completely agree
3.11	If another university would offer me a better study environment, I would consider it wrong to leave the University of Cologne.	Don't agree at all	☐	☐	☐	☐	☐	Completely agree
3.12	It is not reasonable nowadays to participate voluntarily in university committees or organizations.	Don't agree at all	☐	☐	☐	☐	☐	Completely agree

4. Contacts in and outside of university

4.1 **Do you have contact with German students at the university?** ☐ yes ☐ no

4.2 If yes, upon which occasion?

4.3 and how often? ☐ daily/several times a week ☐ once a week ☐ infrequently

4.4 **Do you have contact with other international students at the university?** ☐ yes ☐ no

4.5 If yes, upon which occasion?

4.6 and how often? ☐ daily/several times a week ☐ once a week ☐ infrequently

4.7 **Do you have contact with German students that don't attend your university?** ☐ yes ☐ no

4.8 If yes, upon which occasion?

MUSTER

4.9	and how often?	☐ daily/more times a week	☐ once a week	☐ infrequently
4.10	**Do you have frequent contact with other people or groups outside of university?**	☐ yes	☐ no	

MUSTER

4. Contacts in and outside of university [continued]

4.11 If yes, with whom and upon which occasion?

4.12 And how often?

- ☐ daily/ several times a week
- ☐ once a week
- ☐ infrequently

5. Consultation, Counseling and Orientation at University

Did you have contact with (…) outside of orientation classes to get information and/or counseling? If so, how satisfied were you with the service provided? Is there a reason for your assessment?

5.1 **International Office**

very satisfied ☐ ☐ ☐ ☐ not satisfied at all ☐ not utilized

5.2 With which division?

5.3 Reason for assessment

5.4 **Faculty**

very satisfied ☐ ☐ ☐ ☐ not satisfied at all ☐ not utilized

5.5 With which disivion?

5.6 Reason for assessment

5.7 **Center for International Relations**

very satisfied ☐ ☐ ☐ ☐ not satisfied at all ☐ not utilized

5.8 With which disivion?

5.9 Reason for assessment

5.10 **Studentenwerk**

very satisfied ☐ ☐ ☐ ☐ not satisfied at all ☐ not utilized

5.11 With which division?

MUSTER

5.12 Reason for assessment

MUSTER

5. Consultation, Counseling and Orientation at University [Continued]

5.13 **Student's Council** very satisfied ☐ ☐ ☐ ☐ not satisfied at all ☐ not utilized

5.14 Reason for assessment

5.15 **Other** very satisfied ☐ ☐ ☐ ☐ not satisfied at all ☐ not utilized

5.16 With which disivion?

5.17 Reason for assessment

6. Finances and Employment

6.1 How do you make a living? (multiple answers possible)

☐ Work ☐ Parents/ Family ☐ grand

☐ Savings ☐ other

7. Contents of Studienstart International

7. German Classes: How useful did you find…

7.1 Erasmus useful ☐ ☐ ☐ not useful

7.2 German for physicians useful not useful

7.3 Tutorial Terminology useful ☐ ☐ ☐ not useful

7.4 other German classes useful not useful

Studienstart International Classes: How useful did you find….?

7.5 Intercultural awareness useful ☐ ☐ ☐ not useful

7.6 Study Competencies useful not useful

7.7 Orientation Classes useful ☐ ☐ ☐ not useful

8.**Professional Studies**

8. Professional Studies [continued]

8.1 How many credits did you receive this semester?

8.2 Do you feel well-prepared for your further course of studies? ☐ yes ☐ no

8.3 If not, what are you still missing?

8.4 Do you know where to turn to when questions during your course of studies arise? ☐ yes ☐ no

8.5 Did you make contacts to your fellow students in Studienstart International during this semester you would like to continue in the future? ☐ yes ☐ no

8.6 What did you like especially about Studienstart International?

8.7 What didn't you like about Studienstart International?

8.8 Did you face some serious difficulties this semester?

8.9 Do you have practical ideas for improvement?

9. Thank you for your participation!
